# Function and mechanism of mesenchymal stem cells in the healing of diabetic foot wounds

**DOI:** 10.3389/fendo.2023.1099310

**Published:** 2023-03-16

**Authors:** Xiaoping Yu, Pan Liu, Zheng Li, Zhengdong Zhang

**Affiliations:** ^1^ School of Medicine and Nursing, Chengdu University, Chengdu, Sichuan, China; ^2^ Hospital of Chengdu University of Traditional Chinese Medicine, Chengdu, Sichuan, China; ^3^ People’s Hospital of Jiulongpo District, Chongqing, China; ^4^ School of Clinical Medicine, Chengdu Medical College, Chengdu, Sichuan, China; ^5^ Department of Orthopedics, The First Affiliated Hospital of Chengdu Medical College, Chengdu, Sichuan, China

**Keywords:** Diabetic foot, Wound healing, Mesenchymal stem cells, Angiogenesis, mechanism

## Abstract

Diabetes has become a global public health problem. Diabetic foot is one of the most severe complications of diabetes, which often places a heavy economic burden on patients and seriously affects their quality of life. The current conventional treatment for the diabetic foot can only relieve the symptoms or delay the progression of the disease but cannot repair damaged blood vessels and nerves. An increasing number of studies have shown that mesenchymal stem cells (MSCs) can promote angiogenesis and re-epithelialization, participate in immune regulation, reduce inflammation, and finally repair diabetic foot ulcer (DFU), rendering it an effective means of treating diabetic foot disease. Currently, stem cells used in the treatment of diabetic foot are divided into two categories: autologous and allogeneic. They are mainly derived from the bone marrow, umbilical cord, adipose tissue, and placenta. MSCs from different sources have similar characteristics and subtle differences. Mastering their features to better select and use MSCs is the premise of improving the therapeutic effect of DFU. This article reviews the types and characteristics of MSCs and their molecular mechanisms and functions in treating DFU to provide innovative ideas for using MSCs to treat diabetic foot and promote wound healing.

## 1 Introduction

Diabetes is a significant global public health problem (1). The number of diabetic patients in 2021 was 536.6 million, and it is expected to increase to approximately 783.2 million people by 2045 (2). With the prolongation and aggravation of the disease, patients with diabetes often present with severe lower extremity vascular disease, leading to DFU. Diabetic foot is one of the most severe complications of diabetes and is the leading cause of surgical non-traumatic amputation (3). Studies have found that approximately 25% of people with diabetes will suffer a DFU in their lifetime, and 30% of people with a diabetic foot will experience disease progression that would eventually leads to amputation (4, 5).

Currently, there is no effective clinical treatment plan for diabetic foot, as conservative medical treatment is only a routine method for diabetic foot treatment. For patients with severe ischemia and unsatisfactory effects of systemic drug treatment, vascular intervention and other operations are necessary to implement blood-flow reconstruction. However, in patients with a diabetic foot, the distal vascular outflow tract is poor, and vascular lesions of the lower extremities are diffuse and multiple. Vascular intervention can only improve stenosis of large vessels to a certain extent, and the improvement effect is limited. Studies have reported that patients with diabetic feet are prone to restenosis after the intervention, the recovery rate of peripheral blood flow is still very low, and the amputation rate is still high (6). Diabetic foot is becoming a worldwide public health problem threatening human health (7, 8). Therefore, a new method to accelerate diabetic wound healing is urgently required.

Previous studies have shown that approximately 50% of diabetic foot cases are caused by neuropathy alone, while peripheral arterial occlusive disease accounts for only 15% of cases. Furthermore, in 35% of cases, diabetic foot is caused by a combination of neuropathy and vascular disease (9, 10). In addition, microvascular diseases, biomechanical abnormalities, joint activity, and infection are increased, and multiple causes can interact (11). As a result, peripheral disease, neuropathy, deformity, previous amputation, and infection are the main factors that lead to DFU development (12).. Currently, conventional treatments—including wound dressing, hyperbaric oxygen therapy (HBOT), negative pressure wound therapy, total contact casting bracing, and wound debridement—can only relieve patients’ symptoms or delay the disease progression. However, they cannot repair damaged blood vessels and nerves. An increasing number of studies have shown that MSCs can promote angiogenesis and re-epithelialization, participate in immune regulation, reduce inflammation, and finally repair DFU, rendering it an effective means of treating diabetic foot disease (13); it is a potential new method for the treatment of the diabetic foot. This article reviews stem cells’ function and molecular mechanisms in treating diabetic foot, to provide innovative ideas for using stem cells to treat diabetic foot and promote wound healing.

## 2 Pathogenesis of DFU

Various factors cause the formation of DFU, and the common causes are poor blood sugar control, neuropathy, ischemia, nutritional dysfunction, trauma, and local infection, among others. The advanced glycation end products (AGEs) is a general term for a series of highly active end products formed by non-enzymatic glycosylation (also known as Maillard reaction) between the amino groups of proteins, fatty acids or nucleic acids, and the aldehyde groups of reducing sugars, which is highly associated with the complications of diabetes (14). In diabetic patients, due to metabolic disorders, chronic inflammation and accumulation of AGEs, vascular endothelial injury and hyperplasia, enhanced platelet adhesion, micro-thrombosis, microvascular bleeding, and exudation occur (15). In addition to abnormal glucose metabolism, diabetic patients are often accompanied by abnormal lipid metabolism, which promotes the release of inflammatory mediators, thus inducing the infiltration of macrophages and other immune cells (16). High lipid and sugar promotes the generation of inflammatory mediators, ultimately leading to sustained high inflammation in the body (17). In diabetic patients, the phagocytosis function of white blood cells and related immune cells is down-regulated. The duration of inflammatory factors in diabetic foot ulcer wounds is prolonged to compensate for the decline in white blood cell activity, leading to the downregulated function of fibroblasts and vascular endothelial cells. The formation of granulation tissue is inhibited (18, 19). Under the stimulation of a high glucose environment, the oxidative stress level of the body increases, and a high level of reactive oxygen species (ROS) will lead to the weakened antioxidant effect of the body, and inhibit the release of cytokines and growth factors and the formation of fibroblasts, collagen fibers, and new blood vessels (20, 21). Finally, capillary stenosis or obstruction exacerbates microcirculation disturbance.

Furthermore, metabolic disorders of diabetes lead to degeneration of peripheral nerve axons and nerve membrane cells, motor, sensory, and autonomic nerves dysfunction, resulting in further decline of limb perfusion effect, sensory dysfunction, muscle atrophy, and tendon and ligament sclerosis (22), followed by foot deformities and increased pressure on the forefoot. Metabolic products cannot be excluded, while extremal ischemia and hypoxia, bacterial growth, extremal ulceration, wound healing is challenging, and foot infection can become worsened (23). As blood flow is impaired, it is often difficult for drugs to reach the affected area, and DFU can progress from a simple infection to widespread gangrene (24). The occurrence and development of DFU involve various pathophysiological processes, and these complex processes often transform and superimpose each other, which renders the treatment of DFU a challenge.

## 3 Conventional treatment for DFU

Since the occurrence of DFU, people have been looking for the best treatment method. Conventional treatments of DFU mainly include wound debridement, wound dressing, hyperbaric oxygen therapy, negative pressure wound therapy, and off-loading.

Debridement is the most commonly used method, and the widely used types include surgical debridement, enzyme debridement, biological debridement, and ultrasonic debridement (25). The clearance goals include removing deactivated, necrotic, and infected tissue from the ulcer and retaining healthy, blood supply-rich tissue. In addition, debridement promotes healing through the surrounding healthy granulation tissue by eliminating infected tissue, senescent cells, and bacterial biofilms (26). Debridement is the most basic method in the treatment of DFU.

Negative pressure wound therapy involves placing a vacuum device on the ulcer wound after debridement. This vacuum device can collect large amounts of exudate, keep the wound clean and dry, and reduce the frequency of dressing replacement (27). In addition, continuous negative pressure drainage can also provide an irrigation solution to promote wound healing.

Hyperbaric Oxygen Therapy (HBOT) can be divided into two methods: local delivery of oxygen to ulcers and systemic delivery of oxygen. HBOT can improve local tissue perfusion, stimulate collagen synthesis, growth factor production, and neovascularization (28). In DFU patients, local oxygenation of ulcers is impaired. HBOT can also inhibit anaerobic bacteria and reduce the use of antibiotics (29, 30). However, the therapeutic value of HBOT obtained through clinical studies remains controversial. Some studies have suggested that HBOT could improve short-term but not long-term ulcer healing efficacy of DFU and could not reduce the amputation rate of DUF (31, 32).

The primary function of wound dressing is to provide a protective barrier for DFU. Meanwhile, some new bandages can inhibit bacteria and promote the speed of blood vessel and tissue regeneration (33). Hydrogels and alginate are currently used for medical dressings, and silver ions and other nanoparticles can significantly improve the therapeutic effect (34–36). For example, Tsang et al. reported that dressing containing nanocrystalline silver and manuka honey could effectively play an antibacterial role in treating DFU and inhibit the generation of drug-resistant bacteria (37). Wound dressing for various sources is constantly being improved and developed.

Shear stress and vertical pressure on the plantar as the ground surface are adverse factors for DFU healing (38). Therefore, the principle of offloading is to reduce pressure on the plantar and forefoot of the DFU (39). The several ways to relieve foot load include orthopedic walking aids and modified shoes used in DFU treatment (40). Compared with the modified shoes, the total contact casting bracing can reduce the load on the sole, mechanically help to reduce and redistribute the pressure of the DFU, and contribute to the repair of ulcers, and is considered an important means for the treatment of DFU (41, 42). However, the production of total contact casting bracing requires personalization for different patients.

Other considerations, such as glycemic control, vascular assessment, use of sensitive antibiotics, and psychotherapy in patients with DFU, have been fully considered in previous research (43, 44). In addition, amputation may be a life-saving option if the patient’s condition becomes too severe to salvage a limb (45, 46). Although there are many therapeutic methods, treating DFU is still one of the thorny problems in the complications of diabetes.

## 4 MSCs and stem cells

MSCs are a type of pluripotent stem cells that were first discovered by FriedenStein et al. (47, 48). The term “mesenchymal” refers to the embryonic origin of cells. “Mesenchymal stem cells” were initially named fibroblast colony-forming units or bone marrow stromal cells, and can differentiate into various mesodermal tissues (49). The mesoderm is one of the three main layers formed early in embryonic development. It produces various connective tissues, such as muscle, bone, cartilage, and fat, and cells forming blood vessels, blood cells, and the urogenital system (50). In addition, it has been found that MSCs can be used as ectoderm and endoderm-derived cells, such as liver and nerve cells (51). The differentiation potential of MSCs may depend on the source of stem cells, amplification conditions, and the culture microenvironment. The differentiation process can be induced by specific hormones, growth factors, or specific differentiation agents (52). A complex interaction of genetic and epigenetic factors also controls the differentiation process. Genetic factors include the expression of particular transcription factors and signaling molecules, while epigenetic factors include histone modification, DNA methylation, and altered expression of non-coding RNA (53).

The main feature of stem cells is their diverse origin and potential for self-renewal and multi-differentiation. Moreover, MSCs promote tissue repair by releasing growth factors and cytokines, which help recruit other cells to the damaged site (54). These growth factors and cytokines also promote the formation of new blood vessels necessary for tissue repair. MSCs can also regulate immune system activity, reduce inflammation, and suppress immune responses (55), rendering stem cell therapy a new option for repairing and regenerating tissues. This property renders them promising candidates for cellular therapies for a variety of diseases, such as autoimmune diseases and graft-versus-host diseases.

Numerous studies have found that stem cell transplantation can improve various diseases, such as diabetic retinopathy and keratopathy (56, 57), congenital cataracts (58), ocular surface burns (59, 60), severe skin burns (61, 62), myocardial infarction (63, 64), Parkinson’s disease (65, 66), Huntington’s disease (67, 68), and DFU (48, 69). In addition, MSCs can promote wound healing (70, 71) and serve as a cell source for many tissue engineering applications, including bone regeneration (72, 73), cartilage regeneration (74, 75), neurogenesis (76, 77), myocardial regeneration (78, 79), inflammatory bowel disease (80) and DFU (81, 82).

MSCs are easy to obtain and they belong to a class of immunodeficient cells. In general, allogeneic gene transplantation does not cause immune rejection. Previous studies have shown that most stem cells express low levels of human leukocyte antigen (HLA) class I. They do not express or lower express HLA class II, nor do they express co-stimulator factor (CD40, CD80, and CD86) and surface markers of hematopoietic cells (CD34, CD45, CD79, and CD14) (83–85). This property enables stem cells to be immune-privileged without causing immunological conflict between host and transplanted cells (86). The presence of HLA class I is important because low levels of HLA class I can protect cells from natural killer (NK) cell-mediated cytotoxicity (87). It has been reported that MSCs express HLA class II after being exposed to the pro-inflammatory microenvironment of damaged tissues (86). MSCs have been reported to be highly immunogenic after transplantation into the host (88). More than 90% of undifferentiated MSCS express HLA class II when exposed to IFN-γ (89). In addition, Agudo et al. reported that Hair follicle stem cells downregulate major histocompatibility complex (MHC) class I in the static state to avoid immune surveillance (90). Changes in the immunogenicity of MSCs may depend on many factors, including cell state and microenvironment. Therefore, more studies on the details related to the immunogenicity of MSCs are needed to help improve the efficiency of MSCs transplantation.

Compared with mononuclear cells and endothelial progenitor cells mainly derived from autologous cells, they are suitable for a wide range of clinical applications and the promotion of later stem cell products. MSCs express a series of cell surface immune markers, based on which the International Society for Cellular Therapy (ISCT) formulated a set of identification criteria for MSCs in 2006 (1): plasticity and adherence (2); expression of CD73, CD90, and CD105, and no expression of CD14, CD34, CD45, CD11b, CD79α, CD19, and HLA-DR; (3) capability to differentiate into chondrocytes, osteoblasts, and adipocytes (91). The ISCT guidelines aim to standardize mesenchymal stem cell research and promote collaboration among investigators. Generally, MSCs from different tissue sources can express the typical immunophenotypes of MSCs, but there are slight differences in the expression of the remaining immunophenotypes. It is possible that this standard will be revised in the future as research progresses and new knowledge becomes available.

### 4.1 Types of MSCs

There are many sources of MSCs. Current research shows that stem cells can be extracted from different tissues. There are more studies on bone marrow MSCs (BM-MSCs), human numerical core MSCs (hUC-MSCs), adipose tissue-derived MSCs (ADSCs), urine-derived stem cells (USCs), and placenta-derived MSCs (PD-MSCs).

BM-MSCs are a group of heterogeneous cells composed of pluripotent adult stem cells with the potential ability for multi-differentiation, including chondrocytic, adipocytic, or osteocytic lineages (92). It represents ~ 0.001–0.01% of bone marrow mononuclear cells (BMMNCs) and expresses CD73, CD90, and CD105 but does not express CD14, CD45, CD34, or CD11b, CD79α, CD19, or HLA-DR surface molecules (93). Due to its low abundance, extensive *in vitro* culture and amplification are required to obtain sufficient quantities for research or clinical use (94). The acquisition process of BM-MSCs is often invasive and costly. In addition, the cell quality of BM-MSCs decreased significantly with the increase in donor age.

Human umbilical cord MSCs (hUC-MSCs) were separated from Wharton’s Jelly, a colloidal tissue surrounding the umbilical cord blood canal (95). It is usually discarded during childbirth; thus, the collection is non-invasive and poses few ethical problems (96). It has the characteristics of a short doubling time (97), long survival time (98), and strong anti-inflammatory ability (99), and long-term *in vitro* culture has little influence on its phenotype and genetic stability (100). Compared with BM-MSCs, hUC-MSCs have a higher proliferative ability and lower expression of HLA-ABC and HLA-DR (101).

Adipose tissue-derived MSCs (ADSCs) are rich in tissue sources. It can be obtained by minimally invasive surgery from subcutaneous white adipose tissue separated from the abdomen, thighs, or buttocks/buttocks of animals or humans (102). The isolation of ADSCs is simple, with high yield (~ 100 mL can be collected from 1000 mL adipose tissue) (103). It can differentiate in multiple lineages, including chondrogenesis, osteogenesis, cardiomyocyte, adipogenesis, neurogenic, and hepatic differentiation (104, 105). ADSCs often express CD34 in low-passage cultures, but this decreases with continuous cell passage (106, 107). Unlike BM-MSCs, ASCs do not express the sialoglycoprotein podocalyxin (PODXL) or the adhesion marker CD106 (108, 109).

Tissue sources of placenta-derived MSCs (PD-MSCs) include amniotic fluid, amniotic membrane, chorionic plate, chorionic villi, decidua basalis, complete placenta, and complete placenta (110). Stem cell-like cells in the placenta have higher differentiation potential and self-renewal ability than other tissue-derived MSCs (111). In addition, it has shown low immune properties *in vitro* and *in vivo* studies (112). PD-MSCs have also been shown to enhance the differentiation of monocytes from inflammatory M1 macrophages to M2-like macrophages (113), suggesting that PD-MSCs have the potential to improve inflammatory diseases. However, MSCS isolated from different parts of the placenta have different subtle properties. For example, the placental tissue comprises two separate individual tissues (the maternal placental tissues and the fetal). MSCs derived from fetal placental tissues have significantly stronger proliferative capacity than those derived from maternal placental tissues (114). To understand their different characteristics for better use in future research, more research data are needed to clarify the accuracy of their data further.

Zhang et al., in 2008, first identified a urine stem cell population and found that it could expand over ten generations *in vitro* (115). This stem cell population was named urine-derived stem cells (USCs). USCs are easier to obtain than MSCs. They can be extracted directly from excreted urine and are non-invasive, painless, and low-cost (116). It has the same characteristics as those of USCs isolated from the upper urinary tract. It was found that USCs showed normal karyotypes regardless of passage (117, 118). USCs can differentiate into bone, cartilage, and adipose lineages, as well as urothelial cells, smooth muscle cells, endothelial cells, kidney cells, and podocytes, showing the potential for multidirectional differentiation (119–122). USCs expressed several MSCs markers, including CD44, CD73, and vimentin (123), and also expressed adhesion markers such as CD29 and CD166, but not CD31 (124, 125). It was reported that no teratoma was formed when USCs were injected into immunodeficient mice, showing an absence of the tumorigenic phenotype (126).

Gingival mesenchymal stem cells (GMSCs) can be obtained from periodontal tissue, gingival ligaments, and dental pulp. Similar to MSCs from other sources, GMSCs have MSCs-related cell surfaces markers such as CD73, CD90, CD105, and stromal cell antigen 1 (STRO-1) (127). In addition, studies have shown that GMSCs not only have the potential to differentiate into three lines of mesoderm (adipocytes, osteocytes, and chondrocytes) but can also transdifferentiate into ectoderm and endoderm cell lineages, such as keratinocytes, endothelial cells, and nerve cells (128, 129). In addition, GMSCs also have an anti-inflammatory function and immunomodulatory ability (130, 131), and can promote the differentiation of macrophages (132). Furthermore, GMSCS are homogenous, rapidly proliferating, and not tumorigenic, and have stable morphological and functional characteristics under higher passage (130).

Recently, scientists isolated mixed cell populations with mesenchymal and epithelial features from normal human labial minor salivary glands (133). Subsequently, it was confirmed that human labial gland-derived MSCs (LGMSCs) existed in the lamina propria of the oral mucosa (134). Wang et al. successfully isolated MSCs from adult female salivary gland cysts, identifying their characteristic MSCs expression markers, including CD29, CD44, CD73, CD90, and CD105, using flow cytometry. However, the CD34, CD45, CD106, CD117, and the salivary gland epithelium markers (CD49f) were also negative (135). LGMSCs have the potential for osteogenic and lipogenic differentiation, and their ability to differentiate into salivary gland epithelioid‐like cells is stronger than that of other MSCs. However, its adipogenic differentiation ability is lower than that of ADSCs (136, 137). In addition, LGMSCs have the characteristics of a shallow glandular location, are easy to obtain, expand *in vitro*, and regulate immune function (138–140).

In addition, MSCs derived from tissues such as the pancreas and the liver are being explored, which will provide options for multi-source pathways of MSCs in the future. It should be noted that MSCs from type 1 diabetes mellitus (T1DM) donors are similar in phenotype and function to healthy donors. They can maintain normal immunomodulatory or secretory functions (141). However, MSCs from type 2 diabetes mellitus (T2DM) donors often show increased apoptosis and senescence, as well as decreased angiogenesis potential (142).

According to the source of MSCs, those used for treating DFU can be divided into autologous and allogeneic MSCs. Due to the different biological characteristics of MSCs from different tissue sources, their therapeutic mechanisms, adapted diseases, preferred lesions, and effects are also different. Furthermore, the methods used to culture MSCs in different laboratories (including enzyme digestion or tissue-advanced methods) are also different (143). Therefore, the quality and degree of cell expansion are different, and the study results may differ. Consequently, it is necessary to establish a quality control system for MSCs to ensure the stability and effectiveness of MSCs.

### 4.2 Route of administration for MSC therapy

MSCs are mainly used for the treatment of diabetic foot by local delivery and systemic delivery. Local delivery is divided into topical application, topical injection, scaffold, and gel, systemic delivery is divided into intravenous and arterial administration (13). Previous research has shown that BM-MSCs are most effective by intramuscular injection (144), and the best effect of PD-MSCs was obtained by intraperitoneal injection (145).

Yan et al. found that local injection and intravenous infusion of stem cells were used to treat T2DM rat ulcer models, and both administration methods significantly accelerated wound healing. Moreover, systemic administration also had the potential to ameliorate hyperglycemia (146). However, it has been proposed that MSCs be delivered through the whole body, and most of the cells remain in the lungs, with only a small percentage of the cells moving to the ulcer site (147). In addition, intradermal injection of MSCs into the edge of the ulcer significantly improved the wound healing process. However, local injection of MSCs has the disadvantages of poor cell localization, difficult control of cell density and spacing, and impaired cell vitality due to the influence of local wounds (148, 149).

Furthermore, when MSCs are injected locally into the lesion using a syringe, irreversible damage can be caused to the cell membrane, resulting in decreased cell viability (150). For DFU patients with microvascular complications or arterial occlusion, arterial administration often fails to transport MSCs well to the ulcer site, thus affecting the therapeutic effect. When MSCs are administered to the muscle near the lesion site, the muscle tissue can provide oxygen and nutrients to the injected cells, which contributes to the survival of MSCs and improves their function (148). However, the characteristics of MSCs mean their external preparations are difficult. Therefore, it has been proposed to use scaffolds loaded with MSCs as the primary cell carriers to deliver MSCs, to provide a favorable microenvironment for cell attachment, proliferation, differentiation, and guiding host cell migration, to achieve better healing effects (2). Assi et al. found that compared with the control group with an ordinary injection of MSCs, Rolled collagen scaffolds containing MSCs showed better healing ability and increased vascular endothelial growth factor (VEGF) expression and capillary density in the local ulcers; they found increased numbers of fibroblasts, macrophages, and smooth muscle cells (151).

Assis et al. reported an approach to induce angiogenesis using vascular-inducing devices (VIDs) composed of MSCs derived from healthy donors and decellularized lung-derived micro-fragments. These VIDs express and transcribe the entire library of angiogenic factors in a controlled release manner, induce proliferation of fibroblasts and endothelial cells, and induce local vascular network formation within a week after implantation of non-obese diabetic/severe combined immunodeficiency mice (152). They then transplanted the acellular micro-fragment from the bone marrow of an elderly diabetic patient suffering from lower extremity arterial disease and DFU. They found that the MSCs expressed and secreted angiogenic factors similar to those extracted from healthy individuals (153). This provides a good idea for researching and developing stem cells and scaffolds.

**Figure 1:**
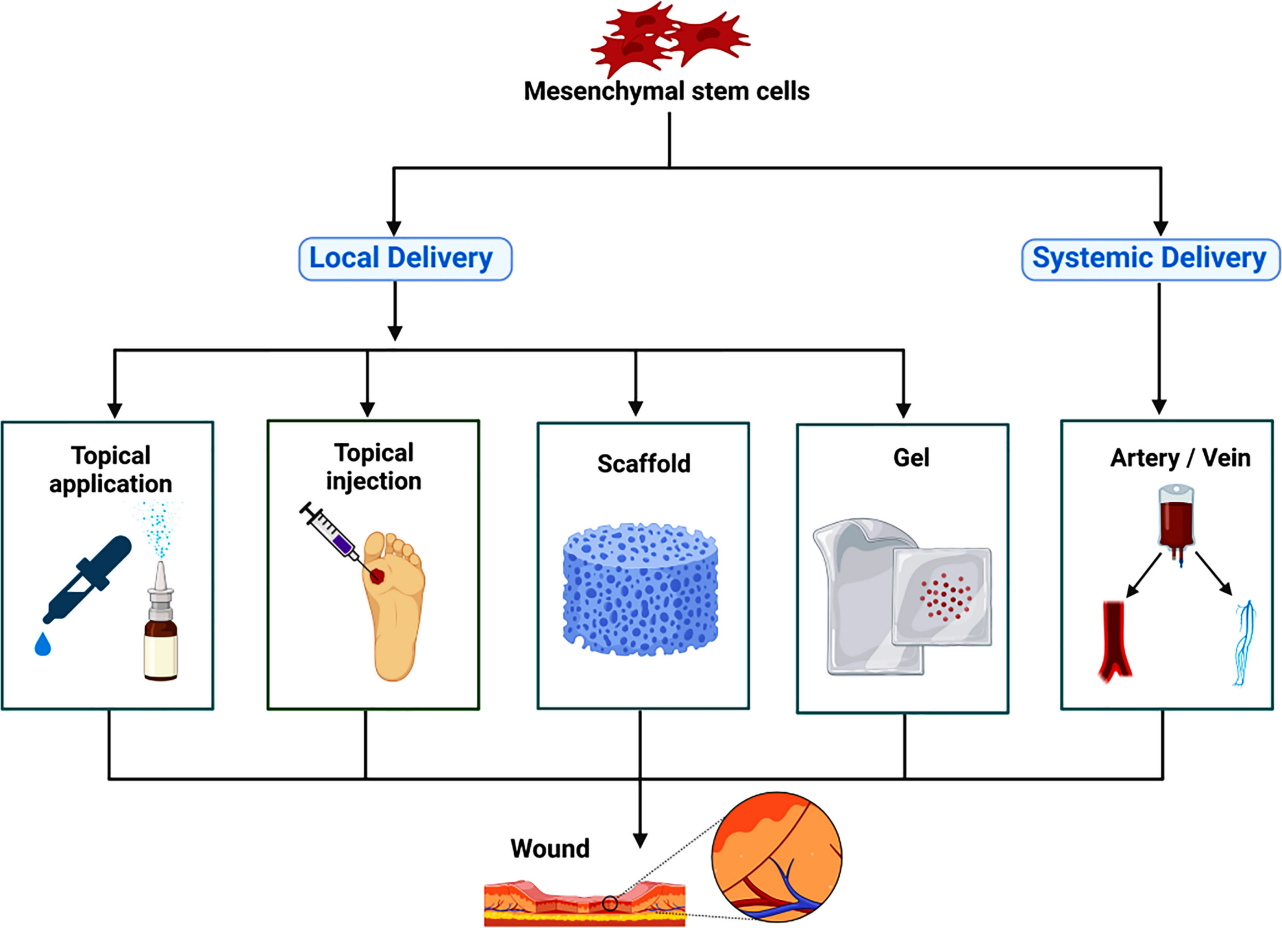
The route of administration for mesenchymal stem cells therapy. Mesenchymal stem cells are mainly used for the treatment of diabetic foot by local delivery and systemic delivery. Local delivery is divided into topical application, topical injection, scaffold, gel and so on; systemic delivery is divided into intravenous administration and arterial administration.

A large number of studies have been devoted to developing excipients that can provide support for MSCs, such as 3D printed collagen, chitosan, polyurethane scaffolds, and cell gels (13, 154, 155), to improve the effective maintenance time for topical application preparations of MSCs (**Figure 1**). In the actual treatment process, we can choose the most appropriate drug administration route by personalized treatment according to the actual condition of patients and the allocation of medical resources.

### 4.3 Mechanisms of MSCs in the treatment of diabetic foot

Cell proliferation, differentiation, and migration are crucial for the physiological processes of DFU wound repair and growth. Wounds result from living tissue damage, and coordinating wound repair is initiated immediately upon damage to the tissue surface. During repair, growth factors and cytokines stimulate signal regulation and coordinate intercellular and intracellular signaling to promote cell proliferation, differentiation, migration, and protein synthesis. Recent studies have shown that various growth factors and molecular mechanisms play a vital role in the occurrence and development of DFU (156, 157).

#### 4.3.1 MSCs can provide a variety of growth factors to promote angiogenesis

One of the essential reasons for diabetic foot secondary to diabetes is the damage and lesions of blood vessels. The formation and regeneration of new blood vessels in the DFU area provide nutrients for the growth of granulation tissue. Therefore, it is especially important for shrinking ulcers and promoting repair. Studies have shown that MSCs can secrete a variety of cytokines, including VEGF, basic fibroblast growth factor, stromal cell-derived factor-1 (SDF-1), keratinocyte growth factor 2, insulin-like growth factor 1, placental growth factor, and epidermal growth factor (EGF). These factors can promote angiogenesis, enhance microhemodynamics, and promote wound healing (158, 159). Among a series of factors regulating angiogenesis and repair, VEGF is the most potent (160).

Shen et al. showed that BM-MSCs could accelerate wound healing in the feet of diabetic mice by improving the activation of vascular endothelial cells and inducing angiogenesis by the paracrine VEGF and other vasoactive factors (161). After transplanting BM-MSCs into diabetic rat foot wounds, Wan et al. found that the expression of VEGF in wound tissue and angiogenesis was increased, which positively affected wound healing in diabetic rats (144). Furthermore, Badillo et al. showed that Mouse liver-derived MSCs increase local growth factor secretion, such as EGF, VEGF, and SDF-1, thus promoting neovascularization, enhancing wound cell recruitment, and improving wound contraction (162). Moreover, BM-MSCs can significantly promote the secretion of key growth factors, such as EGF and VEGF, for repairing and regenerating damaged tissues. They can increase collagen (types I–V) to promote wound healing in diabetic rats (163). Furthermore, Diao et al. demonstrated that in addition to directly promoting angiogenesis, VEGF can activate transcription factors to regulate endothelial progenitor cells (EPCs), recruit EPCs to the bone marrow, and inhibit the apoptosis of EPCs from promoting wound healing (164). These studies suggest that MSCs may directly or indirectly promote angiogenesis at the injury site *via* paracrine growth factors, improve blood flow, and promote the healing of diabetic foot wounds.

#### 4.3.2 MSCs can promote keratinocytes to participate in wound epidermis formation and regulate the local microenvironment

*In vitro* studies have shown that MSCs can differentiate into epidermal cells and function as epidermal cells through different induction methods (165, 166). Kato et al. treated the foot wounds of diabetic rats and control rats with BM-MSCs. They found that the reduced phosphorylated focal adhesion kinase levels were restored when human keratinocytes were cultured in a BM-MSCs-conditioned medium containing high glucose. In addition, the levels of matrix metalloproteinase-2, EGF, and insulin-like growth factor 1 were increased, suggesting that BM-MSCs could promote wound healing in diabetic foot model rats by improving keratinocyte function (167). Additionally, BM-MSCs-treated wounds promote the proliferation of keratinocytes and endothelial cells and promote the migration of macrophages, keratinocytes, and endothelial cells into the wounds of model mice, thereby promoting wound healing (168). Wu et al. used genetically diabetic db/db mice to conduct research and found that VEGF, Angiopoietin-1, and keratinocyte-specific protein keratin were higher in wounds treated with BMSCs. Furthermore, Bmscs significantly promoted the growth of keratinocytes at the wound site, stimulated the formation of new blood vessels, promoted epithelial regeneration at wound sites, and accelerated wound healing (169).

Furthermore, hUC-MSCs can specifically localize to the target ulcer tissue in a rat model of diabetic foot ulcer, promote the secretion of cytokeratin 19, stimulate the formation of keratinocytes and extracellular matrix, and promote epithelial regeneration in ulcerated tissues (170). Although numerous studies have confirmed that MSCs can differentiate into keratinocytes and endothelial cells, their engraftment effects remain controversial. It has been suggested that, under special circumstances, MSCs differentiate into keratinocytes but do not have the full set of expression markers that keratinocytes have (171). For example, Schneider et al. reported that BM-MSCs were cultured in air-exposed on dermal equivalents consisting of collagen types I and III with dermal fibroblasts; they found that MSCs possessed obvious vitality and three-dimensional epidermis-like growth patterns and possessed markers of early and mature epithelial cells without expression of E-cadherin or pan-cytokeratin (172).

Thus, an appropriate culture environment should be selected to cultivate BMCs to improve the success rate of differentiation. It should be noted that the current *in vivo* studies on MSCs observed by DFU models mainly focus on animal models, and the data volume of human models is still small.

#### 4.3.3 MSCs promote cell migration to wound tissue through chemokine receptors-related signaling pathways

Recent studies have shown that various molecular mechanisms, including cell signaling pathways, play important roles in the pathophysiology and healing processes of diabetic foot (173–175). A protein-serine-threonine kinase (AKT) is a serine/threonine kinase that is an important signaling center for various cellular functions. PI13-dependent AKT activation further affects MSC survival, proliferation, migration, and angiogenesis; this pathway plays a core regulatory role (175). The Notch signaling pathway is a short-range communication sensor that regulates stem cell niche maintenance, such as cell differentiation, cell proliferation, and cell death during the development and renewal of adult tissues (176).

Hou et al. found that the conditioned medium of BM-MSCs accelerated the migration and proliferation of human umbilical vein endothelial cells. These processes were closely related to the AKT signaling pathway and independent of the extracellular signal-regulated kinases (ERK) signaling pathway (177). Jun et al. demonstrated that amniotic fluid-derived MSCs (AF-MSCs) promoted wound closure by increasing angiogenic factors while increasing epidermal cell regeneration, and it accelerated the proliferation and migration of dermal fibroblasts and accelerated wound healing through the transforming growth factor-beta (TGF-β)/SMAD2 and PI3K/AKT signaling pathways under hypoxic conditions (178). Liu et al. reported that SDF-1 and chemokine receptor four play important roles in regulating BM-MSCs to promote DFU healing (179). Interestingly, combined treatment with PRP and rat ADSCs promotes angiogenesis, triggers epidermal stem cell proliferation and recruitment by modulating the Notch pathway, and significantly accelerates the healing of experimentally induced diabetic wounds in rats (180). These phenomena suggest that the Notch signaling pathway may be a new potential therapeutic target for diabetic wounds (181, 182).

#### 4.3.4 MSCs can participate in immune regulation and reduce inflammation and tissue damage

**Figure 2:**
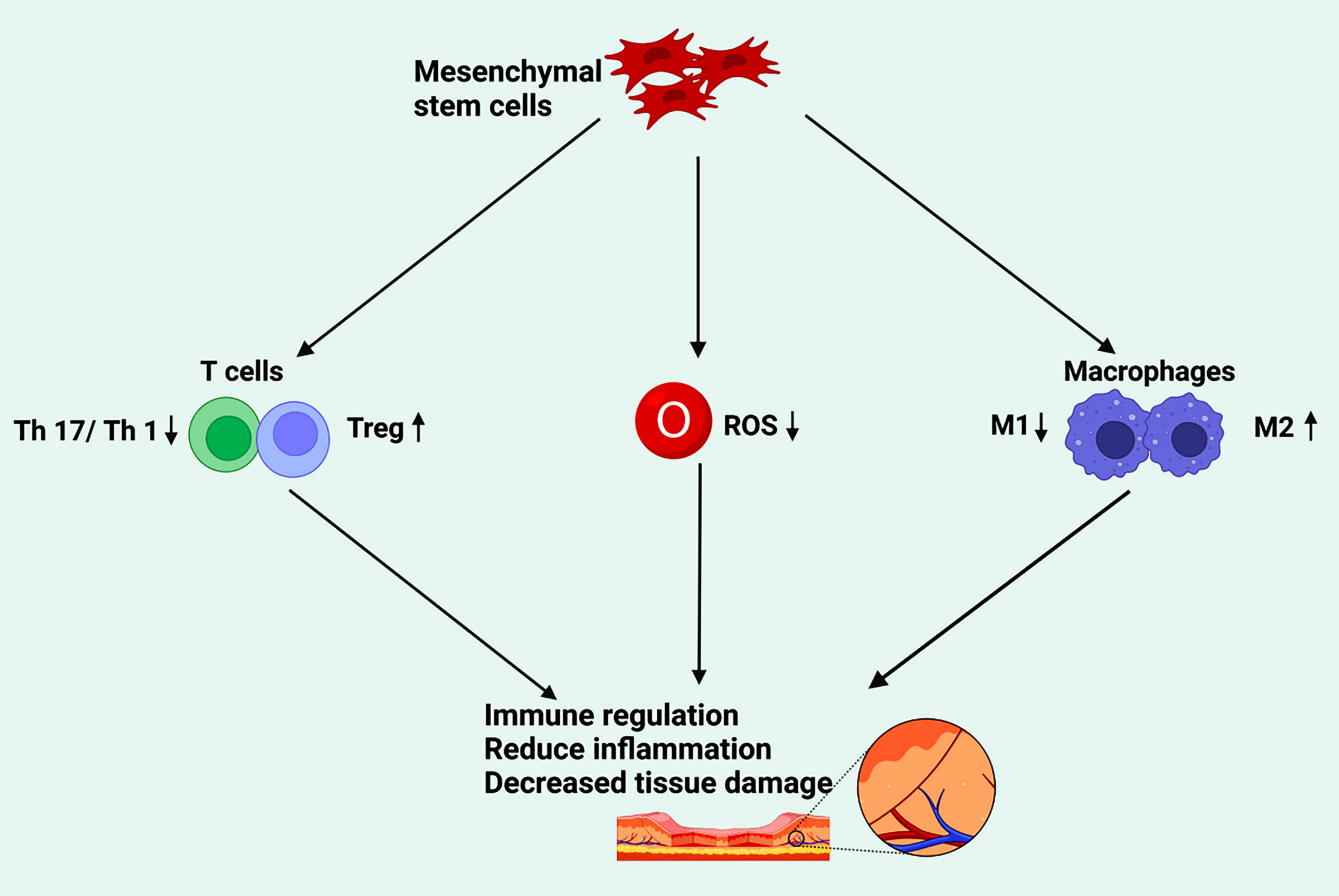
The effect of mesenchymal stem cells on tissue damage through immune regulation. Mesenchymal stem cells participate in immune regulation by inhibiting T17 and T1 cells, promoting Treg cells, downregulating ROS, and accelerating the polarization of M2, so as to reduce inflammation and repair the damage of diabetic foot. (Created in BioRender.com).

In addition to their ability to differentiate into different cell types, MSCs also play a regulatory role in inflammatory and immune responses. Many studies have shown that after cell or tissue injury, MSCs can be activated by inflammatory cytokines and control the process of tissue regeneration by releasing a series of factors that may promote the differentiation and proliferation of progenitor cells while participating in immune regulation and inhibiting inflammatory responses (67, 183, 184). (**Figure 2**).

##### 4.3.4.1 MSCs can modulate immunity by suppressing pro-inflammatory T cells and inducing T regulatory cells

T helper cells 17 (Th17) and T helper cells 1 (Th1) can mediate inflammation (185). CD4+ cells, namely regulatory T cells (Treg), are a subset of specialized immunosuppressive T cells that can specifically express CD25 and CTLA-4 on the cell surface and the transcription factor FoxP3 in the nucleus, which can maintain homeostasis and immune self-tolerance (186) (187).. Li et al. confirmed that 15 patients with diabetic foot disease received hUC-MSC transplantation under insulin treatment, after which blood glucose levels and insulin doses were decreased in all 15 patients. Four weeks after transplantation, CD4^+^CD25 (hi) FoxP3^+^Treg/Th17 and CD4^+^CD25 (hi) FoxP3^+^Treg/Th1 cell ratios increased significantly (p <0.01), while Th17/Th1 cell ratios remained unchanged and VEGF serum levels peaked (188).

##### 4.3.4.2 MSCs can play an immunomodulatory role by reducing the production of reactive oxygen species

ROS are oxygen-free radicals (189, 190). Low ROS levels are beneficial for maintaining cell proliferation, differentiation, and survival, while high ROS levels stimulate immune responses and cause oxidative damage, leading to cell damage and dysfunction (191). When tissues are damaged, phagocytes in the body phagocytose bacteria, apoptotic inflammatory cells, or cell debris to kill pathogens. However, after phagocytosis, long-lived neutrophils generate substantial ROS, causing a respiratory burst that causes tissue damage.

Some studies have suggested that antioxidant activity of MSCs may occur through cell contact or paracrine reduction of lipid peroxidation and protein oxidation (192, 193). MSCs reduce inflammation and oxidative stress in several diseases. These effects include reducing the expression of ROS-producing enzymes myeloperoxidase, inducible nitric oxide synthase, and nitrogen oxides and reducing inflammatory cytokines IL-1β, IL-4, IL-6, IL-9, tumor necrosis factor-alpha (TNF-α), and IFN-γ (194, 195). MSCs can also directly reduce ROS and myeloperoxidase in stimulated monocytes and macrophages, thereby inhibiting their pro-inflammatory phenotypes (196, 197). By enhancing the secretion and expression of stanniocalcin (STC)-1, MSCs significantly inhibited the production of mitochondrial ROS in macrophages, and inhibited nucleotide binding oligomeric domain (NOD)-like receptor pyrin domain containing 3 (NLRP3) inflammasome, Caspase-1 activation, IL-1β production, TNF-α and IL-6 transcription (197). Transplantation of PD-MSCs has also been shown to promote diabetic wound healing by reducing TNF-α, IL-6, and IL-1 pro-inflammatory cytokines and inhibiting NF-κB signal transduction (198). Li et al. showed that mesenchymal stem cell-conditioned medium could reduce the overproduction of ROS in high glucose and/or lipopolysaccharide induced keratinocytes, and reversed the downregulation of mitogen-activated protein kinase (MEK)1/2 and ERK 1/2 phosphorylation induced by high glucose and/or lipopolysaccharide, improving keratinocyte proliferation and migration in diabetes-like microenvironments (199). Raffaghello et al. found that BM-MSCs could prevent excessive or inappropriate oxidative metabolism, activate neutrophils, and inhibit their apoptosis, thereby reducing ROS production without affecting the phagocytic ability of neutrophils (200). Exosomes secreted by human ADSCs can alleviate DFU progression by preventing the senescence of EPCs and inhibiting the expression of ROS and inflammatory cytokines (201).

MSCs play an immunomodulatory role by inhibiting ROS production and enhancing mitochondrial function in macrophages and neutrophils. Therefore, in the future, the role of MSCs in anti-ROS and immune regulation in diseases should be considered to help optimize the therapeutic effect of DFU.

##### 4.3.4.3 MSCs can exert immunomodulatory effects by reducing classically activated M1 macrophages and increasing selectively activated M2 macrophages

M1 macrophages have traditionally been associated with proinflammatory events. M1 macrophages are defined as macrophages that produce proinflammatory cytokines, which mediate resistance to pathogens, and exhibit powerful bactericidal properties, but also cause tissue destruction and inhibit angiogenesis (202, 203). The M1 macrophages are characterized by an enhanced ability to secrete cytokines such as IL-1β, TNF, IL-12, ROS, and IL-18 (204). On the contrary, M2 macrophages are thought to have anti-inflammatory and pro-regenerative effects. The molecules expressed by M2 macrophages include IL-10, Arginase1 (Arg1), resistin-like-α (also called Fizz1), Mrc1 (also called CD206), and chitinase 3-like 3 (also called Ym1) (205). These molecules may be involved in tissue remodeling, parasitic infections, immunomodulatory functions of tumors, and promote angiogenesis (206). They represent the two ends of the macrophage activation spectrum and can transform into each other in specific microenvironments.

In the first stage of ulcer healing, pro-inflammatory M1 macrophages infiltrate the ulcer to remove bacteria, dead cells, and foreign bodies from the ulcer (207). When tissue begins to repair an acute wound, the M1 macrophage population changes to an M2 phenotype, resulting in anti-inflammatory and regenerative effects (208). In chronic wounds, if proinflammatory macrophages persist with the M1 phenotype, the transformation to the M2 anti-inflammatory phenotype is impeded, which leads to impaired tissue repair (209, 210). A persistent high glucose environment *in vivo* stimulates macrophages to secrete pro-inflammatory cytokines, such as TNF-α, IL-1β, IL-6 and ROS, leading to a vicious cycle of persistent M1 macrophage phenotypes and a persistently higher state of inflammation in DFU (211). Therefore, it can be inferred that M2 macrophages can promote the healing of DFU. Thus, transforming M1 macrophages into adequate M2 macrophages in the wound-healing process of DFU may be an effective therapeutic idea.

Dayan et al. proposed that co-culture of human BM-MSCs and hUC-MSCs with macrophages reduced the overall macrophage/monocyte levels, including decreased pro-inflammatory M1 macrophages. In contrast, the level of alternately activated anti-inflammatory M2 macrophages was significantly increased (212). In addition, human GMSCs can induce M2 polarization of macrophages to play an immunomodulatory role, thereby enhancing wound repair (213). Yu et al. found that rat ADSCs reduce the number of M1 macrophages and increase the number of CD163 (+) M2 macrophages, delaying the progression of diabetes and its complications (214). Chen et al. used 3D nanofiber scaffolds loaded with mouse BM-MSCs to act on the wounds of diabetic mice. The ratio of alternately activated M2/classically activated M1 macrophages was significantly increased, promoting wound healing in diabetic mice (215). PGE2 secreted by hUC-MSCs rescues endothelial cell dysfunction and improves the local microenvironment of vascular endothelial cells IL-10 and VEGF. It improves angiogenesis to promote wound healing by regulating M1-to-M2 macrophage polarization in diabetic wounds (216).

These reports on the promotion of the polarization of M1 macrophages into M2 macrophages and promotion of the healing of DFU have brought good news to patients; however, they need to be further studied.

#### 4.3.5 MSCs/MSCs-derived exosomes can promote ischemic tissue repair and angiogenesis in the diabetic foot *via* microRNA

With the rapid development of “cell-free therapy,” MSC-derived small extracellular vesicles (EVs) have become a research hotspot for treating various diseases. Exosomes are the smallest extracellular vesicles in the range of 30–150 nm in diameter, with a bilayer structure and disc-like morphology. They mediate signal transduction between adjacent cells, distant cells, and organs by delivering noncoding RNAs, proteins, and DNA (217). Chen et al. found that TNF-α, interleukin 6 (IL-6), and vascular cell adhesion molecule 1 (VCAM-1) induced heterogeneous secretion of exosomes from MSCs. Furthermore, they defined a novel pro-angiogenic miRNA by RNA sequencing, miRNA-21-5p, a novel mechanism and novel biomarker by which exosomes can promote angiogenesis and ischemia tissue repair in DFU (218). In contrast, Chen et al. found that TNF-α and IL-6 down-regulated angiogenic-related miRNA in MSCs-exo, suggesting that the angiogenesis potential of MSCs-exo decreased after TNF-α and IL6 stimulation (219). Moreover, MSCs-EVs can upregulate the expression of the VEGF gene through miRNA-210-3p and activate key pro-angiogenic proteins, such as ERK and AKT, to improve microcirculation and promote angiogenesis (220). In addition, BM-MSCs downregulate the target genes TRAF6 and IRAK1 through exosomal miR-146a, reducing the expression of NF-κB, IL-6, and MIP-2, thereby inhibiting the inflammatory response and promoting the repair of diabetic wounds (221). Finally, Yu et al. demonstrated that BM-MSCs-derived exosomes enhanced the biological function of endothelial cells through the exosomal miRNA-221-3p-mediated AKT/eNOS pathway, thereby promoting the repair of diabetic wounds (222). This suggests that MSCs-EVs can promote angiogenesis and wound healing in treating DFU, but inflammatory factors may inhibit the potential of MSCs-EVs to promote angiogenesis.

**Figure 3:**
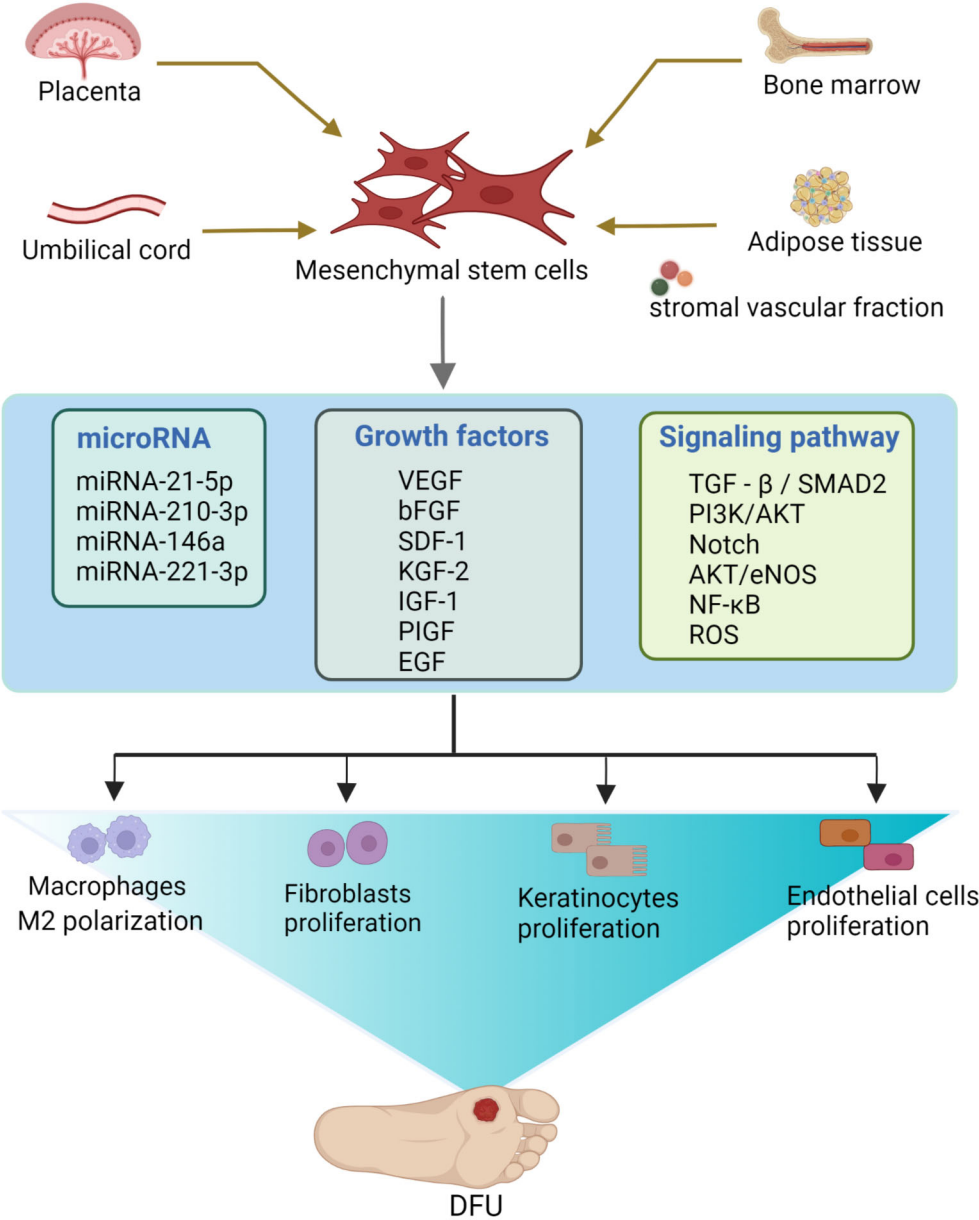
The therapeutic mechanism of mesenchymal stem cells (MSCs) for diabetic foot. MSCs are mainly derived from bone marrow, umbilical cord, adipose tissue, placenta, and other parts. During the treatment of diabetic foot, mesenchymal stem cells repair diabetic foot mainly through the proliferation of fibroblasts, keratinocytes and endothelial cells, as well as angiogenesis and polarization of macrophage M2. In this process, paracrine growth factors, related signaling pathways and microRNAs are involved. (Created in BioRender.com).

In conclusion, MSCs can accelerate the repair of diabetic foot wounds by synergistic effects, such as immunomodulation, upregulation of anti-inflammatory factors, or downregulation of pro-inflammatory factors to reduce the inflammatory response, increase blood supply to ulcers, promote granulation tissue formation, stimulate epidermal regeneration (223), and finally increase the limb salvage rate in diabetic foot patients (224, 225) (**Figure 3**, **Table 1**). However, in the context of hyperglycemia and chronic inflammation in DFU patients, AGEs lead to a decline in the survival rate of MSCs and seriously reduce the repair efficiency of MSCs. In addition, inflammatory factors may inhibit the ability of MSCs to promote vascular regeneration and repair. Therefore, good blood glucose control and inflammation control must be considered in treating DUF by MSCs (148, 219, 227). Although the current study has achieved relatively positive clinical results, optimal efficacy still needs to be explored.

**Table 1:** Potential role of MSCs in the healing of diabetic foot.

Stem cell type	Method of administration	Changes at the molecular level	Changes Histology or clinical manifestation	References
BM-MSCs	Topical application	VEGF and NGF ↑	Promote angiogenesis and accelerate wound healing	(161)
BM-MSCs	Intramuscular injection	VEGF ↑	Promote granulation tissue formation and re-epithelialization, enhance angiogenesis, and accelerates wound closure	(144)
Mouse liver-derived MSCs	Topical application	VEGF, EGF, TGFβ-1 and SDF-1α ↑	Improve neovascularization and promote wound contraction	(162)
BM-MSCs	Topical application	TGF-β, KGF, EGFand VEGF ↑, collagen Type I-V ↑	Increase wound breaking strength (WBS) of fascial wounds and accelerate repair of damaged tissue	(163)
BM-MSCs	Topical cell injection	pFAK, MMP2, EGF and IGF-1 ↑	Enhance epithelialization improve delayed wound healing	(167)
BM-MSCs	Subcutaneous injection	EGF, KGF, IGF-1, VEGF-α and EPO ↑	Enhance new blood vessel formation	(168)
BM-MSCs	Topical cell injection	Ang-1 and VEGF-α ↑	Enhance epithelialization and increase angiogenesis	(169)
hUC-MSCs	Left femoral artery injection	Collagen I and III ↑, Cytokeratin 19 ↑	Enhance epithelization and increase granulation tissue	(170)
BM-MSCs	Topical application	VEGF ↑	Accelerate angiogenesis, restore blood supply to wounds and promote wound healing	(161)
ADSCs	Topical cell injection	ß Integrin, Notch, DLL4, Jag1, Hes1 and Hey1 ↑	Enhance angiogenesis	(180)
hUC-MSCs	Intramuscular injection	VEGF ↑, CD4^+^CD25(hi)FoxP3^+^Treg/Th17 and CD4^+^CD25(hi)FoxP3^+^Treg/Th1 ↑	Improve symptoms such as numbness, pain, coldness and intermittent claudication	(188)
PD-MSCs	Subcutaneous injection	TNF-α, IL-6, IL-1 and NF-κB↓	Promote dermal wound healing in a diabetic Goto-Kakizaki rat model	(198)
ADSCs-exos	Topical cell injection	IL-1β, IL-6, TNF-α and ROS ↓	Promote angiogenesis, increase granulation tissue formation and significantly reduce the ulcer area of wounds	(201)
BM-MSCs	Scaffold’s implantation	Formation of M1 macrophages ↓, IL-6 and TNF-α↓, formation of M2 macrophages ↑, IL-4 and IL-10 ↑	Enhance granulation tissue formation, promote angiogenesis and accelerate collagen deposition.	(215)
hUC-MSCs	Subcutaneous injection	M2 macrophages ↑	Increase angiogenesis and accelerate wound healing	(216)
hUC-MSCs-exos	Intramuscular injection	VEGFR, AKT and MAPK ↑	Promote ischemic tissue repair and angiogenesis	(218)
BM-MSCs	Intradermal injection	miRNA-146a ↑, IRAK1, TRAF6, NF-κB, IL-6 and MIP-2↓	Reduce inflammatory response and enhance wound repair	(221)
BM-MSCs-exos	Multipoint injection	miRNA-211-3p, p-AKT and p-eNOS ↑	Increase angiogenesis and accelerate wound regeneration	(222)
hUC-MSCs- exos	Topical application	VEGF and TGFβ-1↑	Accelerate wound closure rate	(226)

MSCs, mesenchymal stem cells; BM-MSCs, bone marrow mesenchymal stem cells; hUC-MSCs, human umbilical cord mesenchymal stem cells; ADSCs, adipose tissue-derived mesenchymal stem cells; ADSCs-exos, exosomes from adipose tissue-derived mesenchymal stem cells; hUC-MSCs-exos, exosomes from human umbilical cord mesenchymal stem cells; BM-MSCs-exos, exosomes from bone marrow mesenchymal stem cells; VEGF, vas-cular endothelial growth factor; NGF, nerve growth factor; EGF, epidermal growth factor; TGFβ-1:transforming growth factor beta-1; SDF-1α,stromal cell-derived factor-1α; TGF-β, transforming growth factor beta; KGF, ker-atinocyte growth factor; pFAK, phosphorylated focal adhesion kinase; MMP2, matrix met-alloproteinase-2; IGF-1, insulin-like growth factor 1; VEGF-α, vas-cular endothelial growth factor-α; EPO, Erythropoietin; Ang-1, Angiopoietin-1; DLL4, Delta-like canonical Notch ligand 4; Hes1, Hairy Enhancer of Split-1; IL-1β, Interleukin-1β; IL-6, Interleukin-6; TNF-α, tumor necrosis factor alpha; ROS, Reactive oxygen species; IL-4, Interleukin-4; IL-10, Interleukin-10; VEGFR, vascular endothelial growth factor receptor; MAPK, mitogen-activated protein kinase; IRAK1, IL-1 receptor-associated kinase 1; TRAF6, TNF receptor-associated factor 6; NF-κB, nuclear factor-κB; MIP-2, macrophage inflammatory protein-2; p-eNOS, phosphorylation-endothelial nitric oxide synthase.“↑” represents up-regulation/increased expression, "↓" represents down-regulation/decreased expression.

### 4.4 MSC-related derivatives

With the continuous in-depth research and elucidation of the mechanism of action, exploring MSCs or other cell derivatives with a more clear mechanism of action for DFU treatment has become a current research hotspot. The use of these derivatives to treat DFU shows efficacy and characteristics similar to those of MSCs. MSCs or cell derivatives reported in related studies include exosomes, exosome gels, conditioned medium, growth factors, platelet lysates, and platelet-rich plasma (PRP). Among these derivatives, research on exosomes is currently hot. Li et al. suggested that AD-MSCs exosomes could significantly improve inflammation in rat DFU wounds and reduce the expression of oxidative stress-related proteins in the wound while promoting tissue regeneration, the proliferation of EPCs, angiogenesis, and growth factor expression (201).

Yang et al. proposed that the efficient delivery and enhanced exosome capacity of hUC-MSCs-derived exosomes in a Pluronic F-127 hydrogel could accelerate diabetic wound healing. Therefore, MSCs-derived exosome therapy may be a new treatment for chronic wound skin regeneration (226). Dash et al. found that autologous implantation of BM-MSCs in patients with diabetic foot accelerated the lower extremity wound healing process and significantly improved clinical symptoms (228). Furthermore, some research has found that injection of an MSC-conditioned medium promotes wound closure in diabetic mice (156, 169). Growth factors promote wound healing in patients with DFU. Transgenic *Lactobacillus merceris* secretes platelet-derived growth factor-BB, a dimeric peptide that binds to platelet-derived growth factor receptors and stimulates cell proliferation and survival. This could be a cost-effective method for patients and be used in regenerative medicine strategies to promote tissue repair (229). The combination of MSCs and PRP has been found to enhance wound healing (230). A human clinical study reported that PRP was significantly better than topical antiseptic dressings in cleaning diabetic ulcers and found healing rates of up to 86%, which significantly improved the 68% healing rate of antimicrobial ointment dressings (231). PRP contains growth factors that promote cell proliferation and matrix synthesis and could be considered a candidate treatment for the nonhealing of DFU (232). In conclusion, MSC-related derivatives are a promising new method for treating DFU; however, their exact efficacy remains to be confirmed by further studies.

### 4.5 MSCS treatment of diabetic foot-related clinical trials

As mentioned above, conventional treatment of DFU has been mentioned. However, conventional treatment is not always effective. For example, patients with DFU have lower limb artery lesions involving the lower leg arteries and may face amputation if serious vascular diseases occur in the affected limb. MSCs promote tissue repair and regeneration by increasing extracellular matrix, repairing cell activity, promoting angiogenesis at ulcer sites, secreting growth factors, and forming new keratinocytes (233, 234). To date, MSCs have become a hot spot for DFU, and more clinical research has been widely carried out.

#### 4.5.1 Autologous stem cells

Transplanting autologous stem cells into DFU enhances ulcer healing and reduces amputation rates. They include BM-BMSCs, peripheral blood mononuclear cells (PBMNCs), BMMNCs, ADSCs, and an adipose tissue-derived stromal vascular fraction (SVF).

Yuyama et al. reported autologous BMMNCs transplantation for angiogenesis in patients with limb ischemia (235). A significant proportion of DFU patients suffer from vascular disease. Claeys et al. proposed that percutaneous partial pressure of oxygen could be used as a predictive parameter of DFU associated with vascular disease (236). Kirana et al. included 22 patients with DFU treated with autologous BMMNCs, which resulted in improved wound healing and transcutaneous pressure of oximetry in the affected limb (237). Xu et al. used recombinant human granulocyte colony-stimulating factor (G-CSF) 5–10 µg/kg/day for the proliferation of BM-MSCs in DFU patients for 4–5 consecutive days to promote their release into peripheral blood and then took peripheral blood MSCs and injected them around or at the bottom of ulcers. After 4 weeks of follow-up, the ulcers gradually healed. Digital subtraction angiography (DSA) detection revealed that abundant collateral circulation was established around the lesions of the DFU (238). Huang et al. also used G-CSF to mobilize PBMNCs in treating patients with DFU accompanied by critical limb ischemia (CLI) and achieved significant clinical effect (239). This provides a reference for the diversity of treatment modalities. G-CSF is a growth factor that stimulates bone marrow and mobilizes EPCs, thereby increasing their numbers to cure DFU (240). Lu et al. conducted clinical trials, in which patients with type 2 diabetic feet were given BM-MSCs, BMMNCs, or normal saline (NS). The results illustrated that in promoting the healing of patients with DFU, the BM-MSCs treatment group could be more effective than the BMMNCs treatment group. They also found that the BMMSCs of diabetic patients secret more VEGF, FGF-2, and angiopoietin-1 than BMMNCS under normoxic and hypoxic conditions. Therefore, they believed that BMMSCS is better than BMMNCs in the local vascular generation (241). Procházka et al. performed clinical trials, dividing 96 CLI and DFU patients into two groups. The first group of patients received local treatment of autologous BM-MSCs, while the second group of patients received the standard treatment of medical care. The results suggested that BM-MSCs local treatment can save 79% of the limbs of CLI and DFU patients. Among the 21% of amputation, lymphocytes and platelet reduction may be potentially pathogenic. The primary amputation rate of the control group is 44%. Experiments confirmed that BM-MSCs can greatly improve the prognosis of DFU and reduce the amputation rate. This study found that the low platelet count and the low VEGF level are related to poor healing in bone marrow concentrate. Low platelet and CD34^+^ cell concentrations were present in most unhealed patients, but moderate platelet and CD34^+^ cell concentrations were present in most healed patients rather than either of the two extremes. However, for patients with low platelet counts, if they are accompanied by VEGF with high local concentration, the wound healing was satisfactory. Most amputations that are still not saved after using autologous BM-MSCS for treatment are secondary infections. These treatments are proposed to emphasize the importance of debridement and anti-infection (242). Scatena et al. treated 38 patients with DFU and no-option critical limb ischemia (NO-CLI) with intramuscular and perifocal injections of PBMNCs. Patients treated with PBMNCs had a significantly lower rate of amputation than those (38 patients) treated with standard care under the International Working Group on the Diabetic Foot (IWGDF) guidelines (243), and 86.6% of patients in the PBMNCs group recovered during the 2-year follow-up, compared with only one patient in the control group. The results showed that PBMNCs significantly reduced the amputation rate of DFU with NO-CLI (244). In a recent meta-analysis of autologous MSCs in treating DFU, it was also reported that BMMNCs were more effective in healing foot ulcers in DFU than repeated percutaneous transluminal angioplasty (245, 246).

Adipose tissue-derived SVF is a heterogeneous cell fraction. They include mesenchymal progenitor/stem cells, T cells, pericytes, endothelial cells, and macrophages (247). It has also been specifically used to treat DFU. Han et al. were the first to use uncultured processed lipoaspirate cell autografts to treat diabetic ulcers to stimulate the diabetic fibroblasts of activity and obtained a 100% cure rate of DFU (248). Carstens et al. used adipose-derived SVF in treating 10 patients with non-reconstructive peripheral vascular disease in DFU and achieved good results. They also followed the patients for 6 years and found five patients still showed a consistent clinical benefit (249, 250). Subsequent studies have shown that adipose tissue-derived SVF can induce neovascularization in ischemic conditions in treating chronic DFU, increased transcutaneous partial oxygen pressure, and cutaneous microvascular blood flow (251, 252).

Among autologous MSCs, BM-MSCs and PBMNCs are the most commonly used cell types in DFU studies. Mobilized PBMNCs are preferred over BM-MSCs because of the ease of collection and the avoidance of pain and anesthesia associated with bone marrow biopsy. The ease of execution and good clinical efficacy of adipose-derived SVF brings a new choice for treating DFU. However, further studies are needed to explore the exact use of adipose-derived SVF.

#### 4.5.2 Allogeneic stem cells

Allogeneic stem cells are isolated from an individual of the same species rather than from the recipient, including pluripotent mesenchymal stromal cells from allogeneic sources such as the placenta, umbilical cord, amniotic membrane (148).

Qin et al. included a group of Fontaine II-V DFU patients (28 patients, 34 limbs) with varying degrees of lower extremity arterial disease treated with intravascular infusion and peri-ulcerative injection of the hUC-MSCs after angioplasty. After 3 months of follow-up, the results showed increased neovascularization at the ulcer, ulcer healing, skin temperature, transcutaneous oxygen tension, the ankle-brachial pressure index, and claudication distance were improved noticeably (253). Their study suggests that hUC-MSCs transplantation after angioplasty is a potentially safe and effective clinical treatment for severe DFU. Moon et al. conducted clinical trials, incorporated 59 patients with diabetic foot ulcers, and randomly distributed them to the hydrogel-based allogeneic ADSCs sheets group (n = 30) or the control group of polyurethane film treatment (n = 29). They observed the closure of wounds in the treatment group and control group at weeks 8 and 12, and the median time for the treatment group and the control group was 28.5 days and 63.0 days, respectively. In the 2-year follow-up study, two subjects had a recurrence 6 months after the stem cell therapy ulcer trial, which was different from the site at the beginning of the previous trial. The recurrence was at the toe tip and the plantar foot, susceptible to stress. Later recovery through therapeutic intervention (254). Therefore, in general, they achieved satisfactory results. In addition, there were no serious adverse events related to the treatment of hydrogel-based allogeneic ASC sheets. Therefore, it is proved that hydrogel-based allogeneic ADSCs sheets may be effective and safe for treating DFU (254). Rodríguez et al. launched clinical trials, allowing 28 patients with DFU patients to accept allogeneic BM-MSCs derivatives (n = 12), BM-MSCs (n = 6), or conventional treatment (PolyMem^®^ dress, Ferris, Fort Worth, TX, USA) (n = 10). They conducted a macro assessment of the wound healing process until the ulcers were closed entirely. As a result, no adverse events were reported. Compared with patients receiving conventional treatment, the wound closure rate of DFU patients treated with allogeneic BM-MSCs derivatives or allogeneic BM-MSCs was higher (255). Uzun et al. reported a study that divided 20 patients with DFU accompanied by chronic ulcers into two groups. Patients in the standard group (10 cases) received standard treatment with sterilization, debridement, and dressing coverage. In the study group (10 cases), in addition to routine disinfection and debridement, allogeneic ADSCs were injected into the dermo-epidermal junction and the entire wound surface using intralesional. The results showed that nine patients in the study group had wound healing, while eight patients in the control group had wound healing. The wound healing time of the study group was 31.0 ± 10.7 days, and the wound healing time of the control group was 54.8 ± 15.0 days. In the end, one patient in the study group and two in the control group had their limbs amputated. Allogenic ADSCs were safe for local injection of DFU ulcers with no significant adverse events (256). Their study showed that allogenic ADSCs have a positive therapeutic effect on chronic ulcers of DFU and are superior to standard conventional therapy.

Through the above studies, we can conclude that MSCS and their derivatives and stents delivering MSCs have achieved optimistic clinical effects in the treatment of DFU. However, the role of post-healing patient care, footwear selection, and health education should be considered in preventing the recurrence of ulcers. Furthermore, most of the above clinical trials have a limitation: the sample volume was relatively small. Therefore, multi-center random clinical trial research is recommended to expand the sample volume effectively to obtain more accurate evidence (**Table 2**).

**Table 2:** MSCs treatment of diabetic foot related clinical trials.

Stem cell type	Method	Participants	Outcomes	Method of administration	References
BM-MSCs	Patients randomized to BM-MSCs along with standard wound dressing or standard wound dressing	24	Accelerate healing process and significantly improve clinical parameters in the treatment group	Topical application	(228)
BMMNCs	Patients randomized to BMMNCs or PBMNCs	47	Significantly improve ABI, transcutaneous oxygen pressure, rest pain and pain-free walking time in the BMMNCs group	Intramuscular injection	(235)
BMMNCs	Patients randomized to BMMNCs or expanded bone marrow cells enriched in CD90+ cells	22	Both kinds of cell transplantation are safe and feasible; Improve microcirculation and complete wound healing	Intramuscular injection or intraarterial infusion	(237)
PBMNCs	Patients randomized to PBMNCs mobilized by G-CSF or Filgrastim	127	Promote the establishment of collateral circulation and improve the ischemic area of the patients	Intramuscular injection	(238)
PBMNCs	Patients randomized to a control group or PBMNCs mobilized by G-CSF	28	Significantly improve angiographic scores in the PBMNCs group	Intramuscular injection	(239)
BM-MSCs	Patients randomized to BM-MSCs, BMMNCs, or NS	41	Promote healing of foot ulcers in the BM-MSCs treatment group	Intramuscular injection	(241)
BM-MSCs	Patients randomized to BM-MSCs or standard medical care	96	Improve the prognosis of DFU and reduce amputation rate in the treatment group	Topical application	(242)
PBMNCs	Patients randomized to PBMNCs or a control group	76	Reduce the amputation rate and improve survival and wound healing in the PBMNCs group	Intramuscular injection	(244)
BMMNCs	Patients randomized to BMMNCs with percutaneous transluminal angioplasty or a control group	54	Improve amputation free survival rate and promote ulcers healing	Intramuscular injection	(246)
Adipose-derived SVF	Single arm study and followed the patients for six years	10	Improve tissue perfusion, neovascularization and ABI	Intramuscular injection	(249, 250)
Adipose-derived SVF	Single arm study	10	Increase transcutaneous partial oxygen pressure and cutaneous microvascular blood flow	Subcutaneous injection	(251)
Adipose-derived SVF	Single arm study (Phase I clinical trial)	63	At 12 months, 50 subjects had 100% DFU healing and 4 subjects had ≥85% healing. Promote vascular repair and/or angiogenesis with a good safety	Topical cell injection	(252)
hUC-MSCs	Patients randomized to hUC-MSCs or a control group	53	Increase neovessels and ulcer completely or gradually heal in the hUC-MSCs group	Endovascular infusion and topical cell injection	(253)
ADSCs	Patients randomized to ADSCs or a control group treated with polyurethane film	59	The wound closure rate was increased and the median wound closure time was shortened in the treatment group	Topical application	(254)
BM-MSCs	Patients randomized to BM-MSCs derivatives, BM-MSCs or conventional treatment (PolyMem dress). (Phase 1/2 clinical trial)	28	The wound closure rate was increased in the treatment group	Intradermal injection	(255)
ADSCs	Patients randomized to ADSCs or a standard group (Phase I/2 safety study)	20	Have a positive therapeutic effect on chronic ulcers of DFU and are superior to conventional standard therapy	Intradermal injection	(256)

ADSCs, adipose tissue-derived mesenchymal stem cells; BM-MSCs, bone marrow mesenchymal stem cells; DFU, diabetic foot ulcer; BMMNCs, bone marrow mononuclear cells; NS, normal saline; ABI, ankle-brachial index; G-CSF, granulocyte colony-stimulating factor; SVF, stromal vascular fraction.

### 4.6 Potential disadvantages of MSCs in diabetic foot treatment

Following previous studies in humans and animals, MSCs have achieved encouraging efficacy in treating DFU (241, 254, 257). However, with an increase in research, from the results of recent clinical studies, common side effects of MSCs in DFU are diarrhea, fever, increased serum creatinine level, urticaria, nausea, and vomiting (258, 259). After the passage of stem cells for many times *in vitro*, the multidirectional differentiation potential and paracrine ability may also be reduced, leading to the decline of clinical effect (234). Embryonic stem cells have strong proliferative ability and low differentiation maturity. The introduction of these cells may cause immune rejection and stimulate tumor formation. Therefore, embryonic stem cells should be avoided from DFU treatment as much as possible (260–262). In addition, it has been reported that increasing the number of stem cells applied locally to improve repair efficiency may also increase tumorigenicity (263). Although there may be some side effects of stem cell therapy for diabetic foot, overall, in animal experiments and human studies, BM-MSCs transplantation has achieved positive results in DFU treatment. MSCs transplantation may be a new method that can be used to treat diabetic foot, but the precise utilization of stem cells to control the local microenvironment of DFU to maximize the healing effect is still unknown.

## 5 Conclusions and prospects

These stem cells, which have clear research results, have a largely positive effect on the treatment of DFU while also having the advantage of being used in combination with other treatments to better exert their effects in the treatment of refractory DFU (234, 264). Although stem cells derived from synovium, urine, amniotic fluid, liver, lung, and gingiva have only been reported in sporadic experiments in the treatment of diabetic foot, these stem cells may still be a potential choice for the treatment of diabetic foot in the future. Researchers can explore the characteristics of MSCs derived from different tissues based on *in vivo* and *in vitro* studies. They are expected to clarify their advantages and disadvantages and elucidate the full impact of their therapeutic effects in future studies.

As the first MSCs to be studied for the treatment of DFU, BM-MSCs are relatively convenient to isolate and extract and have achieved good therapeutic effects in many clinical practice applications. Their safety has also been affirmed. After repeated studies, BM-MSCs may be the best choice for treating diabetic foot (265). However, MSCs also have certain shortcomings; for example, the differentiation potential and proliferation ability of BM-MSCs decrease with age (266), and the repair ability is negatively correlated with the number of cell passages (267). This requires us to standardize stem cell therapy for the diabetic foot in the future to maximize its advantages and minimize its disadvantages. So far, different clinical studies have been launched to evaluate the safety and efficacy of MSCs on DFU (NCT03370874, NCT04464213, NCT05610865, NCT04104451, ChiCTR2000036933). It is crucial to standardize the therapeutic efficacy of MSCs products before initiating clinical trials, and this need is driving efforts to develop improved *in vitro* efficacy assays.

Different MSCs can self-renewal and multi-directional differentiation, which brings hope for the treatment of many intractable diseases, such as Parkinson’s disease, myocardial infarction, and bone defects. It is also expected to obtain gratifying clinical effects in basic and clinical application research on treating diabetic foot. Although stem cell therapy’s efficacy and safety in treating diabetic foot have been preliminarily confirmed, further research is needed regarding the treatment mechanisms, efficacy judgments, individual choices of stem cell source, and promotion norms.

In conclusion, DFU treatment with MSCs is a potential, relatively safe, and effective treatment method, among which BM-MSCs may be an ideal choice. However, in the initial treatment plan of the treatment of DFU, it is necessary to select a certain stem cell to specify the specific treatment method according to the characteristics of each stem cell, such as local applications, meridian transmission, local injection, and intravenous application. This is a key step in obtaining the ideal effect, which is still a considerable challenge facing researchers.

## Author contributions

XY, PL and ZL conceived and drafted the manuscript. XY, PL, ZL and ZZ proofread the manuscript and made revisions. XY, PL and ZZ collected the references. ZZ directed the overall design of the manuscript. All authors read and approved the submitted version. All authors contributed to the article and approved the submitted version.
